# AP endonuclease EXO-3 deficiency causes developmental delay and abnormal vulval organogenesis, Pvl, through DNA glycosylase-initiated checkpoint activation in *Caenorhabditis elegans*

**DOI:** 10.1038/s41598-018-35063-6

**Published:** 2018-11-13

**Authors:** Masahiro Miyaji, Yuichiro Hayashi, Masafumi Funakoshi, Akihiro Tanaka, Qiu-Mei Zhang-Akiyama

**Affiliations:** Laboratory of Stress Response Biology, Graduate School of Science, Kyoto University, Kitashirakawa Oiwake-cho, Sakyo-ku, Kyoto, 606-8502 Japan

## Abstract

AP endonuclease deficiency causes cell death and embryonic lethality in mammals. However, the physiological roles of AP endonucleases in multicellular organisms remain unclear, especially after embryogenesis. Here, we report novel physiological roles of the AP endonuclease EXO-3 from larval to adult stages in *Caenorhabditis elegans*, and elucidated the mechanism of the observed phenotypes due to EXO-3 deficiency. The *exo-3* mutants exhibited developmental delay, whereas the *apn-1* mutants did not. The delay depended on the DNA glycosylase NTH-1 and checkpoint kinase CHK-2. The *exo-3* mutants had further developmental delay when treated with AP site-generating agents such as methyl methane sulfonate and sodium bisulfite. The further delay due to sodium bisulfite was dependent on the DNA glycosylase UNG-1. The *exo-3* mutants also demonstrated an increase in *dut-1 (RNAi*)-induced abnormal vulval organogenesis protruding vulva (Pvl), whereas the *apn-1* mutants did not. The increase in Pvl was dependent on UNG-1 and CHK-2. Methyl viologen, *ndx-1 (RNAi)* and *ndx-2 (RNAi)* enhanced the incidence of Pvl among *exo-3* mutants only when combined with *dut-1 (RNAi)*. This further increase in Pvl incidence was independent of NTH-1. These results indicate that EXO-3 prevents developmental delay and Pvl in *C*. *elegans*, which are induced via DNA glycosylase-initiated checkpoint activation.

## Introduction

AP endonucleases are enzymes that function in the repair of DNA damage such as apurinic/apyrimidinic (AP) sites or single-strand breaks with 3′ blocked ends (3′-blocked SSB) in DNA^1,2^. AP sites are generated by the spontaneous hydrolysis of purine/pyrimidine bases (depurination/depyrimidination) or through the DNA glycosylase activity of monofunctional DNA glycosylases, which cleave N-glycosidic bonds in DNA^3,4^. Bifunctional DNA glycosylases, which possess both DNA glycosylase activity and AP lyase activity, generate 3′-blocked SSB by cutting N-glycosidic bonds in DNA and subsequently nicking the sugar phosphate backbone at AP sites^1^. Through the process of DNA replication, AP sites and 3′-blocked SSB can cause double-stranded breaks (DSB), which are considered to be the most deleterious form of DNA lesions and can lead to cell death if not repaired properly^5,6^. Therefore, the repair of AP sites and 3′-blocked SSB is important to protect cells and organisms against their adverse effects. AP sites and 3′-blocked SSB are processed by different enzymatic activities of AP endonucleases, AP endonuclease activity and 3′-phosphodiesterase activity, respectively^1,2,7^. Both activities generate SSB with 3′-OH ends, which are required for the subsequent steps of the repair process known as base excision repair (BER), which is carried out by DNA polymerases and DNA ligases^3^.

The *in vivo* roles of AP endonucleases in unicellular organisms have been well studied. Insufficient AP endonuclease activity in unicellular organisms, including *Escherichia coli (E*. *coli)* and *Saccharomyces cerevisiae (S*. *cerevisiae)*, increases the spontaneous mutation frequency and heightens the sensitivity to DNA damaging agents such as methyl methane sulfonate (MMS), hydrogen peroxide (H_2_O_2_) and gamma rays^8,9^. On the other hand, the physiological roles of AP endonucleases in multicellular organisms are unclear. The knockout of *APEX1*, which encodes the main AP endonuclease APEX1 in mice (human APE1 ortholog), and knockdown of zebrafish *APEX1* (*zfAPEX1*) both cause embryonic lethality^10,11^. In addition to DNA repair activity, APEX1 has redox regulation activity, which is well-conserved among APE1 homologs in mammals. Human APE1 knockdown cells exhibit decreased cellular viability, but this decreased viability is reversed by expression of yeast Apn1 protein, which lacks redox regulation activity^12^. zfAPEX1 does not have the cysteine residue required for redox regulation activity^13^. Thus, the embryonic lethality caused by the knockout of mouse *APEX1* or knockdown of *zfAPEX1* is thought to be due to deficient repair activity, and strongly suggests that the DNA repair activity of AP endonucleases plays an essential role in embryogenesis in multicellular organisms. However, their roles after embryogenesis remain unknown.

*Caenorhabditis elegans (C*. *elegans)* is a useful model animal to study the physiological roles of AP endonucleases. Worms deficient in AP endonuclease genes do not exhibit embryonic lethality and can become fertile adults^14^. Therefore, the physiological effects of the lack of AP endonucleases can be assessed throughout life. In *C*. *elegans*, two AP endonucleases, EXO-3 and APN-1, have been identified^15^. EXO-3 and APN-1 are encoded by the *exo-3* and *apn-1* genes, respectively. EXO-3 is a homologue of mammalian APE1, but the redox regulatory domain is not conserved^13^. A series of *in vitro* experiments revealed that both EXO-3 and APN-1 have AP endonuclease activity and 3′-phosphodiesterase activity^16,17^. Malfunction of AP endonucleases causes severe phenotypes in *C*. *elegans*. *Exo-3 (RNAi)* worms have a reduced life-span in a *cep-1* (*C*. *elegans p53* ortholog)-dependent manner^18^. *Exo-3* mutant worms also exhibit a shortened life-span and reduced self-brood size^14^. *Apn-1 (RNAi)* worms have a moderately reduced longevity only when they are exposed to DNA damaging agents^19^. *Apn-1 (RNAi)* worms also demonstrate retardation of the division of the P1 blastomere^19^. Studies conducted thus far have focused on worms before the larval stages or after the adult stages^14,18,19^. However, the contribution of AP endonucleases during the larval to adult stages remains poorly understood.

To investigate the physiological roles of AP endonucleases from the larval to adult stages in *C*. *elegans*, we assessed the impact of AP endonuclease deficiency on development and vulval organogenesis from the larval to adult stages in *C*. *elegans*. We report newly identified phenotypes of *exo-3(tm4374)* mutants: developmental delay and increased *dut-1 (RNAi)*-induced abnormal vulval organogenesis Pvl. We also present evidence that these phenotypes are induced through a common mechanism where DNA glycosylases initiate DNA damage checkpoint activation.

## Results

### The expression level of AP endonucleases increases after the L4 stage

**Figure 1:**
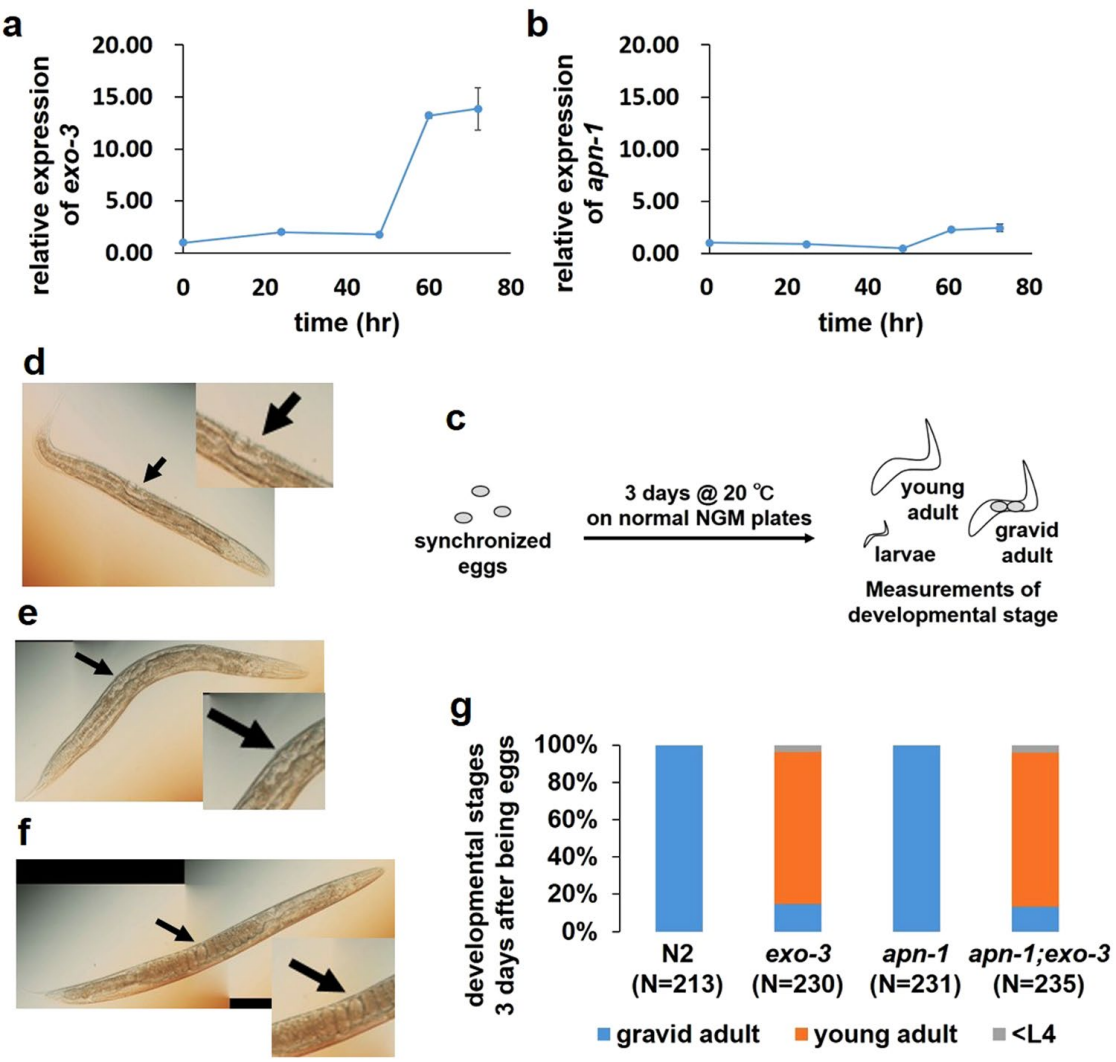
Effects of AP endonuclease deficiency on larval development of worms under normal rearing conditions. (**a**,**b**) At each time point after birth, N2 worms were harvested, and the total RNA isolated from the worms was subjected to real-time PCR analysis using specific primer sets for *exo-3* (**a**) and *apn-1* (**b**). As an internal control, Y45F10D.4 was used. The values represent the mean ± S.E. (n = 3/each time point). (**c**) Experimental scheme. (**d**–**f**) Representative images of worms at each developmental stage. L4 larvae (**d**). Young adults (**e**). Gravid adults (**f**). Black arrows indicate the vulval position. Developmental stages were assessed based on vulval morphology and brooding of eggs. (**g**) Proportion of worms at each developmental stage after 3 days of incubation of fertilized eggs. The values indicate the number of worms at each developmental stage/the number of total surviving worms.

After hatching, *C*. *elegans* develop into adults through four larval stages (L1-L4), each punctuated by molting of the cuticle. Adult stages are still subdivided into two stages: the young adult stage and the gravid adult stage. To gain insight into the role of AP endonucleases after embryogenesis, we measured the temporal change in the mRNA expression level of *exo-3* or *apn-1*. At 0, 24 and 48 hours, when most N2 worms are in the egg stage, L1 stage and L4 stage, respectively, no difference in mRNA expression level was found for both *exo-3* and *apn-1* (Fig. 1a,b). At 60 hours, when most N2 worms are in the young adult stage, the *exo-3* and *apn-1* expression levels were approximately 13-fold and 2.3-fold higher than those at 0 hours, respectively (Fig. 1a,b). The expression level at 72 hours, when most N2 worms are in the gravid adult stage, was the same at 60 hours (Fig. 1a,b). These results suggest that AP endonucleases are required after embryogenesis, especially after the L4 stage.

### The *exo-3* mutants exhibit developmental delay

To clarify the contribution of AP endonucleases to worm development from the L4 to adult stages, worms deficient in either or both AP endonucleases (EXO-3 and APN-1) were incubated under normal rearing conditions for 3 days from the fertilized egg stage (Fig. 1c). Developmental stages among the L4, young adult and gravid adult stages were distinguished by the state of vulval morphology and brooding of eggs (Fig. 1d,f). Although all of the N2 worms developed into gravid adults, only 14% of the *exo-3* mutants were in the gravid adult stage, 85% were in the young adult stage and 1% was in the larval stage (Fig. 1g), suggesting that EXO-3 deficiency causes the cessation of development or developmental delay. In contrast, all of the *apn-1* mutants became gravid adults, and the *apn-1;exo-3* mutants were at almost the same developmental stages as the *exo-3* mutants (Fig. 1g). Twelve hours later, all the *exo-3* and *apn-1;exo-3* mutants reached the gravid adult stage (data not shown), indicating that EXO-3 deficiency does not cause cessation of development at the young adult stage, only developmental delay. To precisely investigate how long the delay of the *exo-3* mutants was, we measured the reaching time to gravid adult of each worm every two hours and calculated the difference of the average time between N2 (N = 8) and the *exo-3* mutants (N = 16). The difference was 6 hours.

### The developmental delay in the exo-3 mutants is dependent on the DNA glycosylase NTH-1

**Figure 2:**
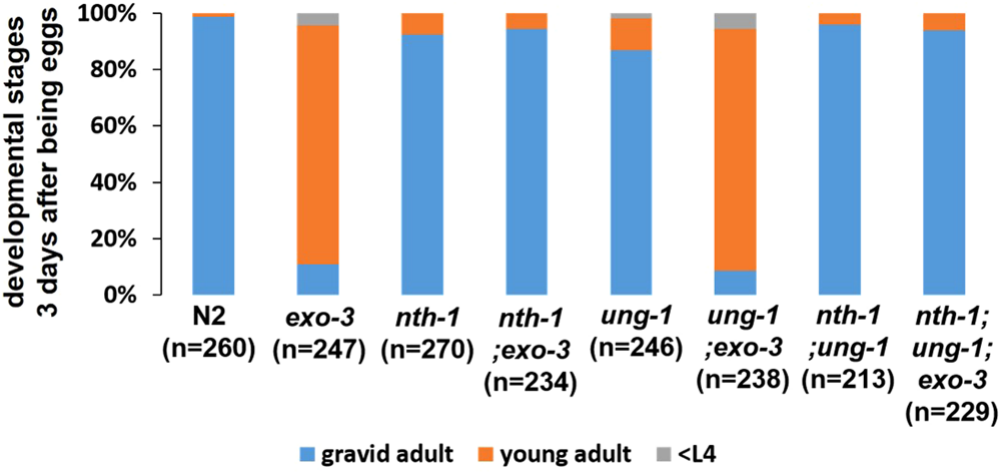
Effects of DNA glycosylase deficiency on larval development of *exo-3 (tm4374)* mutant worms under normal rearing conditions. Proportion of worms at each developmental stage after 3 days of development from eggs. The values indicate the number of worms at each developmental stage/the number of total surviving worms.

It is reasonable to infer that the developmental delay phenotype of *exo-3* mutants is caused by the accumulation of AP sites or 3′-blocked SSB in DNA because these are substrates for EXO-3^15^. These structures in DNA can be generated by DNA glycosylases. Of the two DNA glycosylases conserved in *C*. *elegans*, UNG-1 generates AP sites through monofunctional DNA glycosylase activity on uracil in DNA, and NTH-1 produces 3′-blocked SSB via bifunctional DNA glycosylase activity on oxidative pyrimidine lesions in DNA. Therefore, we examined the dependency of the delay in the *exo-3* mutants on UNG-1 and NTH-1. Three days after developing from eggs, 9% of the *ung-1;exo-3* mutants were in the gravid adult stage, 86% were in the young adult stage and 5% were in the larval stage, and these proportions were almost the same as those shown by the *exo-3* mutants (11% in the gravid adult stage, 85% in the young adult stage and 4% in the larval stage) (Fig. 2), suggesting that the delay is independent of UNG-1. In contrast, 94% of the *nth-1;exo-3* mutants were in the gravid adult stage and 6% were in the young adult stage. The *nth-1;ung-1;exo-3* mutants exhibited similar results (Fig. 2), suggesting that the delay is dependent on NTH-1.

### The developmental delay of the *exo-3* mutants is enhanced by MMS and NaHSO_3_

**Figure 3:**
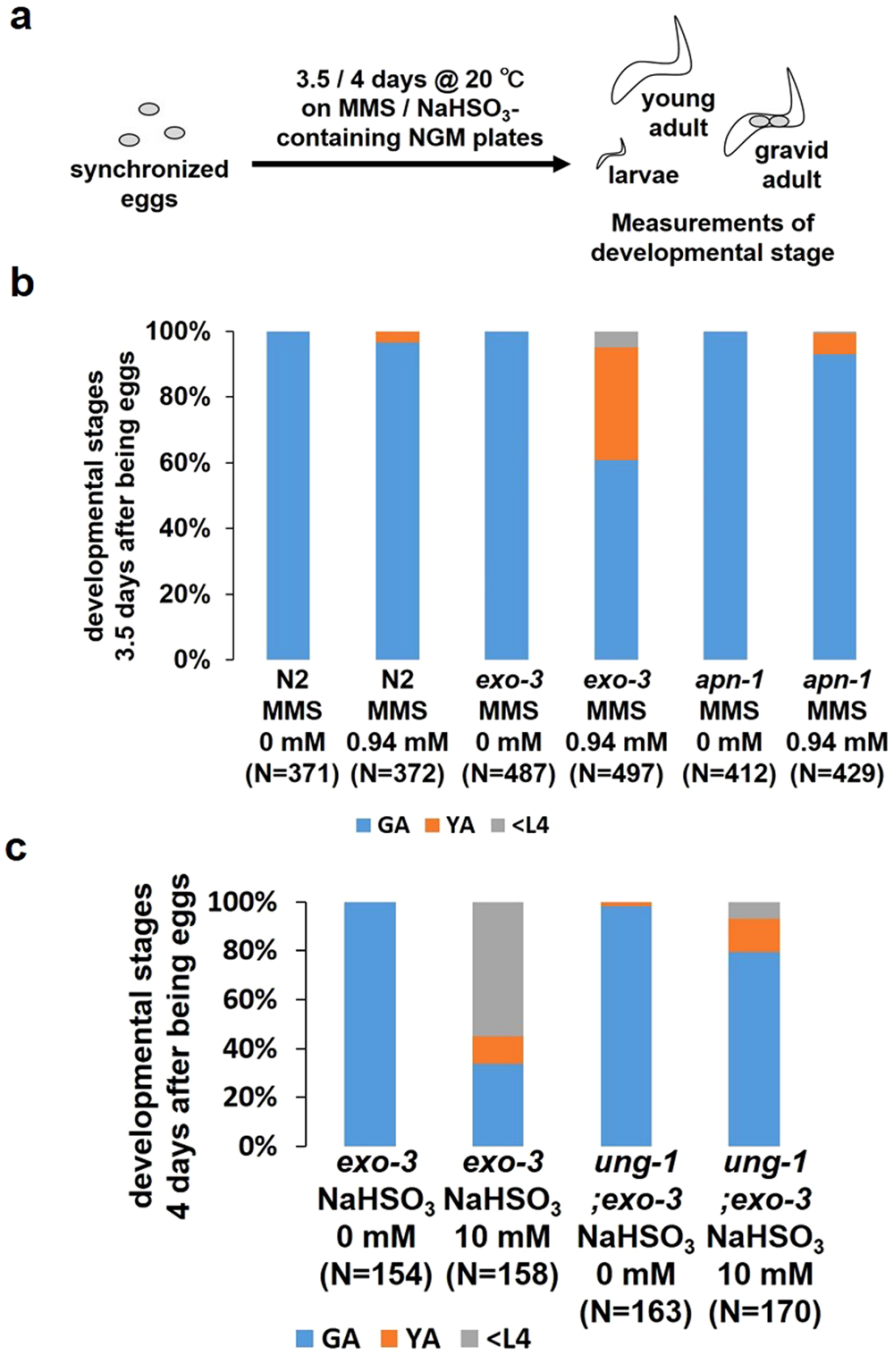
Effects of AP site-generating agents on larval development of worms. (**a**) Experimental scheme. (**b**,**c**) Proportion of worms at each developmental stage 3.5 and 4 days after developing from eggs under 0.94 mM MMS (**b**) and 10 mM NaHSO_3_ (**c**) conditions, respectively. The values indicate the number of worms at each developmental stage/the number of total surviving worms.

To clarify whether AP site-generating agents can cause developmental delay, we conducted a developmental assay using MMS and sodium bisulfite (NaHSO_3_) (Fig. 3a). MMS is known to indirectly create AP sites^20,21^ and shown to induce DNA lesions in the genome of *C*. *elegans*^22^. At 3.5 days after the eggs were laid on plates containing 0.94 mM MMS, 97% of N2 were in the gravid adult stage and 3% were in the larval stage, whereas 61% of the *exo-3* mutants were in the gravid adult stage, 34% were in the young adult stage and 5% were in the larval stage (Fig. 3b), suggesting that MMS-induced AP sites cause further developmental delay in the *exo-3* mutants than in N2. On the other hand, 93% of the *apn-1* mutants were in the gravid adult stage, 6% were in the young adult stage and 1% were in the larval stage, which is similar to the results for N2 (Fig. 3b). NaHSO_3_ damages DNA mainly through deamination of cytosine to uracil^23^. Four days after developing from the egg stage, all *exo-3* mutants not treated with NaHSO_3_ developed into gravid adults, but 33% of those treated with 10 mM NaHSO_3_ were in the gravid adult stage, 12% were in the young adult stage and 55% were in the larval stage (Fig. 3c), indicating that NaHSO_3_ induced developmental delay in the *exo-3* mutants. This delay was alleviated in the *ung-1;exo-3* mutants, as 79% of those treated with 10 mM NaHSO_3_ were in the gravid adult stage, 14% were in the young adult stage and 7% were in the larval stage (Fig. 3c), suggesting that the NaHSO_3_-induced developmental delay was due to UNG-1 activity. Taken together, AP sites may cause developmental delay as well as 3′-blocked SSB generated by NTH-1.

### The developmental delay of the *exo-3* mutants is induced by CHK-2

**Figure 4:**
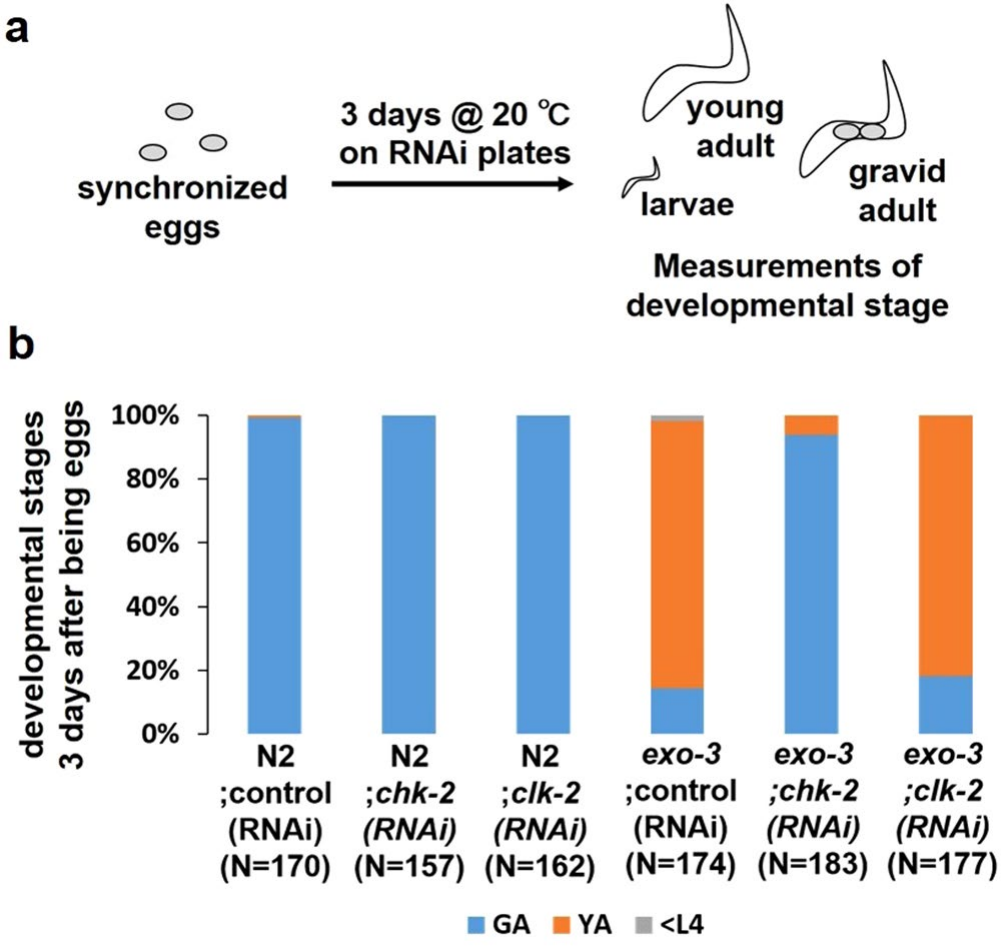
Effects of lack of checkpoint kinases on larval development of *exo-3 (tm4374)* mutant worms. (**a**) Experimental scheme for the developmental assay. (**b**) Proportion of worms at each developmental stage 3 days after development from eggs. The values indicate the number of worms at each developmental stage/the number of total surviving worms.

We next hypothesized that the DNA glycosylase-initiated developmental delay of the *exo-3* mutants was due to DNA damage checkpoint activation driven by cleavage products produced by DNA glycosylases. DNA damage checkpoint genes, such as *chk-2* and *clk-2*, have been described in *C*. *elegans*^24^. To test our hypothesis, the developmental assay was conducted under *chk-2 (RNAi)* or *clk-2 (RNAi)* conditions (Fig. 4a). Although 14% of the *exo-3;control (RNAi)* worms were in the gravid adult stage and 84% were in the young adult stage, and *exo-3;clk-2 (RNAi)* worms demonstrated similar proportions (18% in the gravid adult stage and 82% in the young adult stage), 93% of the *exo-3;chk-2 (RNAi)* worms were in the gravid adult stage (Fig. 4b). Thus, the knockdown of *chk-2* rescued the developmental delay in the *exo-3* mutants, suggesting that CHK-2 induces the developmental delay in the *exo-3* mutants.

### EXO-3 prevents *dut-1 (RNAi)*-induced Pvl formation

**Figure 5:**
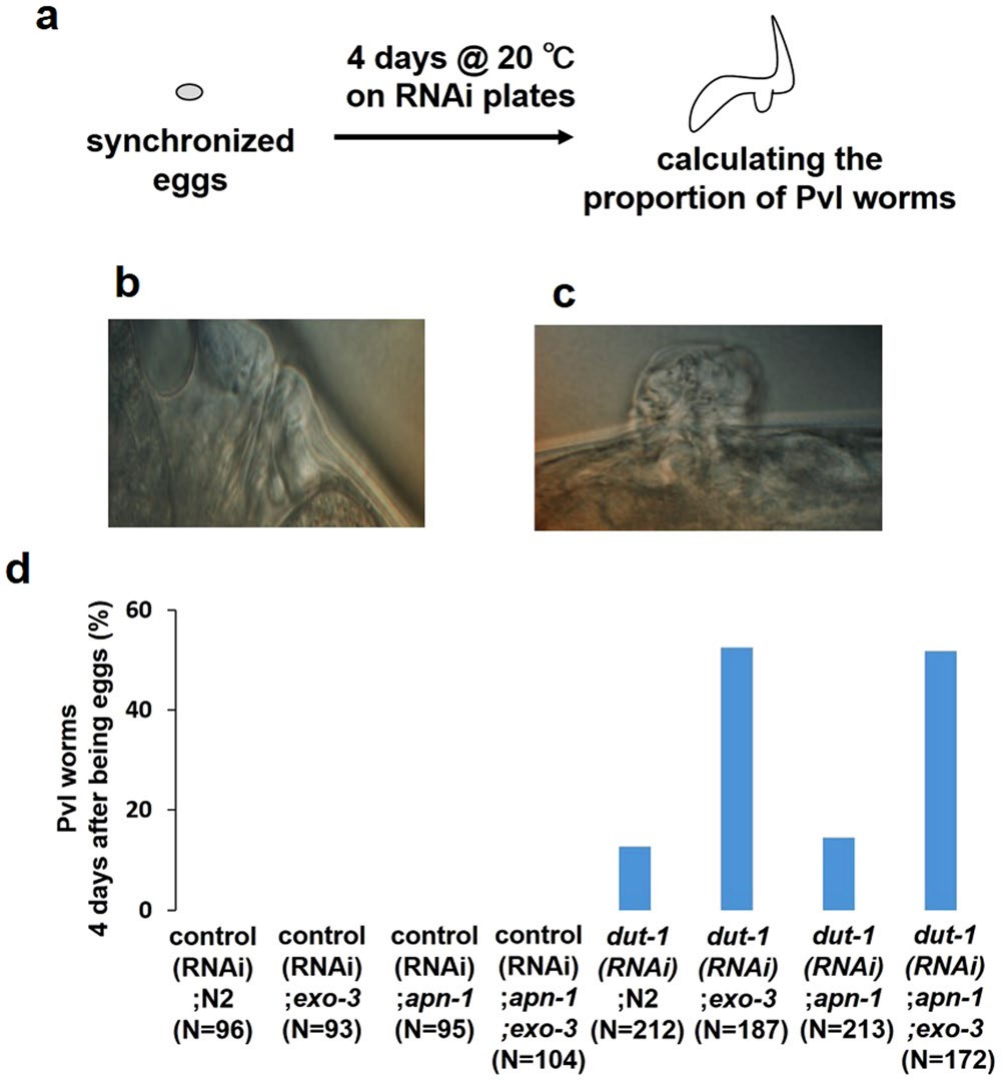
Effects of AP endonuclease deficiency on *dut-1 (RNAi)*-induced Pvl. (**a**) Experimental scheme. (**b**,**c**) Representative images of vulva of control worms (**b**) and *dut-1 (RNAi)*-induced Pvl worms (**c**). (**d**) Proportion of Pvl worms 4 days after development from eggs. The values indicate the number of Pvl worms/the number of adult worms.

DUT-1 is a deoxyuridine triphosphate nucleotidohydrolase (dUTPase), which hydrolyzes dUTP into dUMP. Therefore, *dut-1 (RNAi)* leads to increased dUTP in the nucleotide pool, which is incorporated into DNA through DNA replication instead of dTTP, causing the accumulation of uracil in DNA^25,26^. It has been reported that *dut-1 (RNAi)* induces abnormal vulval organogenesis, Pvl, in wild-type N2 worms, and that the *dut-1 (RNAi)*-induced Pvl is dependent on UNG-1^27^. We speculated that EXO-3 deficiency causes an increase in the incidence of *dut-1 (RNAi)*-induced Pvl because EXO-3 is needed to repair AP sites after the cleavage of uracil in DNA by UNG-1. To confirm this, *dut-1 (RNAi)*-induced Pvl formation was observed in AP endonuclease-deficient mutants (Fig. 5a–c). Of the surviving adults 4 days after developing from eggs, 52% were *exo-3* mutants with *dut-1 (RNAi)*-induced Pvl and 13% were N2 worms with Pvl (Fig. 5d), suggesting that EXO-3 deficiency causes an increase in *dut-1 (RNAi)*-induced Pvl. On the other hand, 15% of the *apn-1* mutants had Pvl, which is similar to the proportion of N2 worms with Pvl (Fig. 5d). The *apn-1;exo-3* mutants and *exo-3* mutants were similar in proportion, with 52% having Pvl (Fig. 5d).

### The increase in *dut-1 (RNAi)*-induced Pvl in the *exo-3* mutants is dependent on UNG-1 irrespective of NTH-1

**Figure 6:**
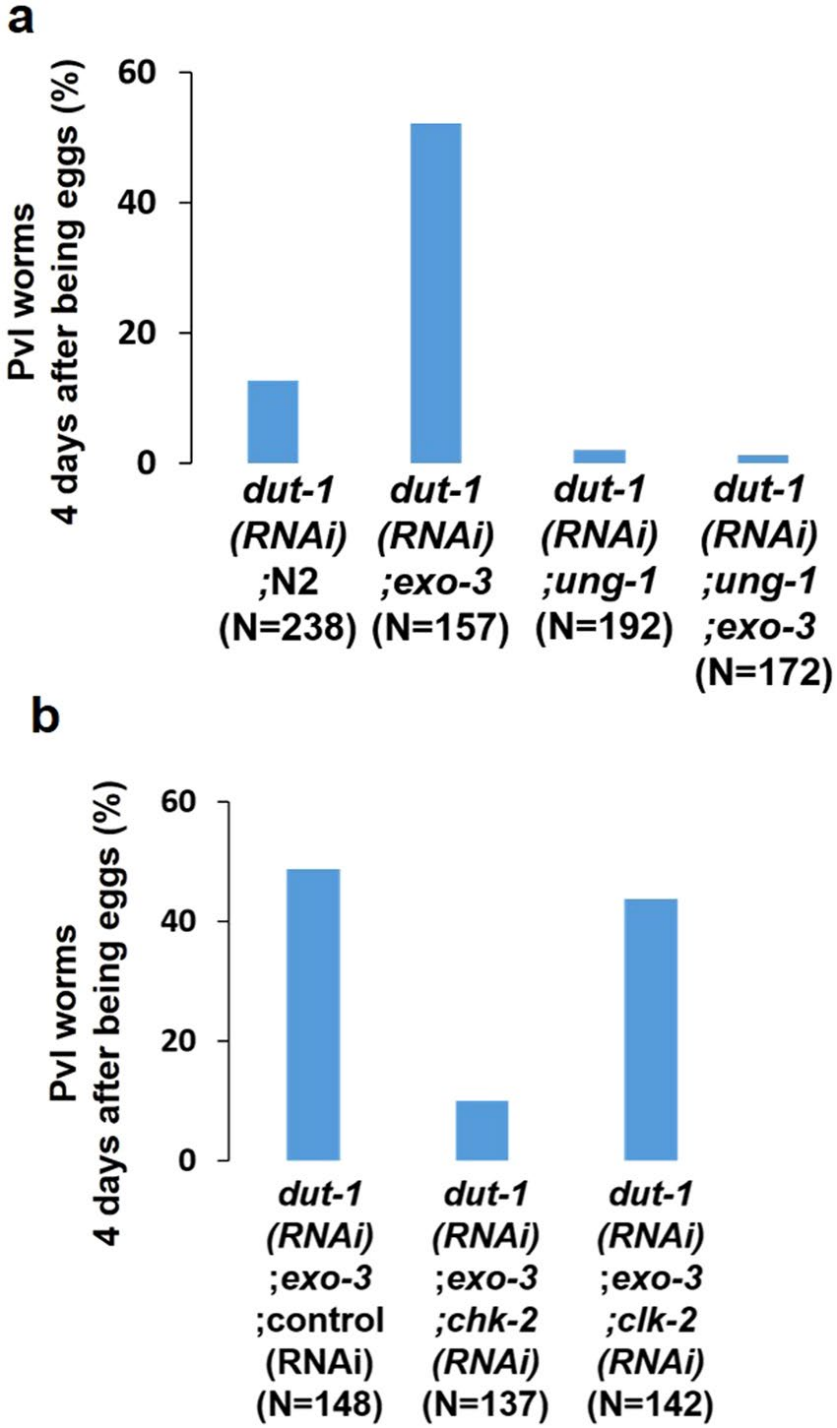
Effects of DNA glycosylase deficiency and lack of checkpoint kinases on *dut-1 (RNAi)*-induced Pvl in *exo-3 (tm4374)* mutant worms. Proportion of Pvl worms 4 days after development from eggs. The values indicate the number of Pvl worms/the number of adult worms. (**a**) Effects of *ung-1 (tm2862)* mutation. (**b**) Effects of *chk-2* (*RNAi)* and *clk-2 (RNAi)*.

To examine whether the increase in *dut-1 (RNAi)*-induced Pvl in the *exo-3* mutants occurred through UNG-1 activity, we investigated the dependency of the phenotype on UNG-1. The percentage of *ung-1;exo-3* mutants with Pvl was 2%, which was the same as that of the *ung-1* mutants with Pvl (1%) (Fig. 6a), suggesting that the increase in Pvl in the *exo-3* mutants is only due to UNG-1 expression.

**Figure 7:**
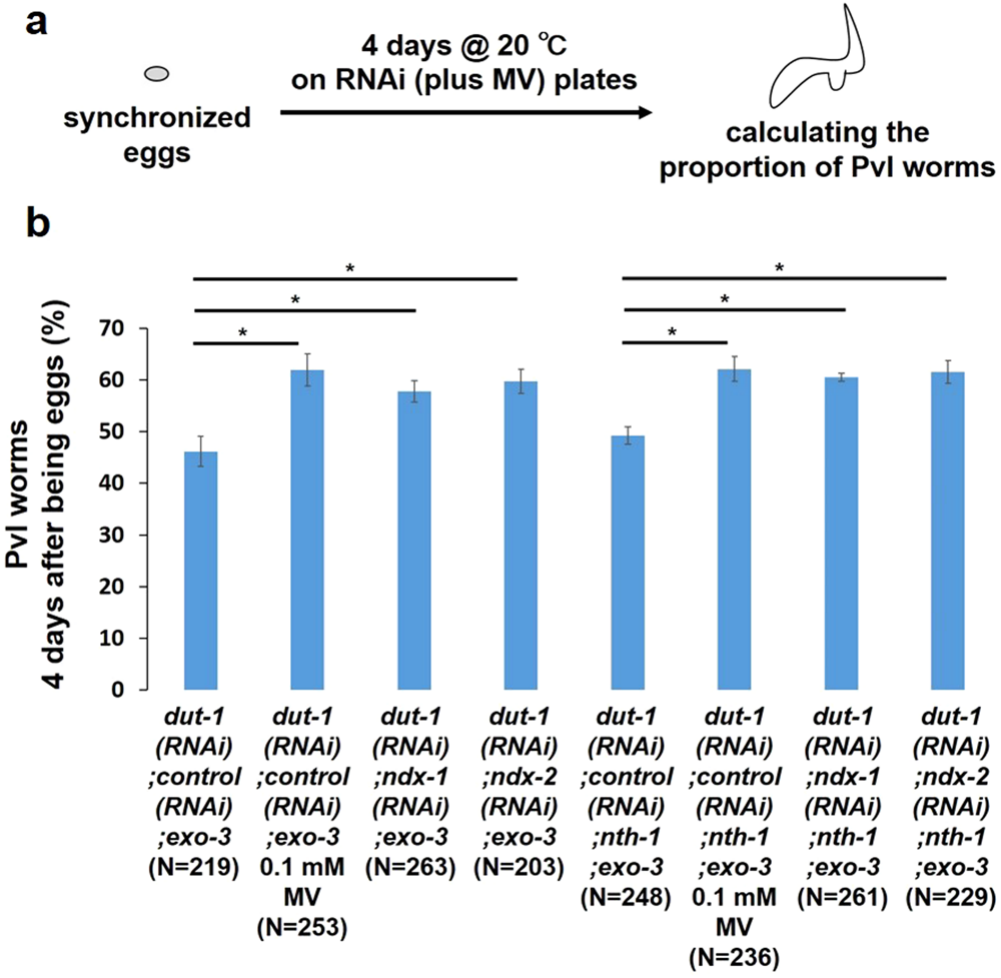
Effects of oxidative DNA damaging agents on *dut-1 (RNAi)*-induced Pvl in *exo-3 (tm4374)* and *exo-3 (tm4374);nth-1 (ok724)* mutant worms by using the double knockdown technique. (**a**) Experimental scheme. (**b**) Proportion of Pvl worms 4 days after development from eggs. The values indicate the number of Pvl worms/the number of adult worms. The values represent the mean ± SD (n = 3). *P < 0.05; one-way ANOVA with Tukey’s test for multiple comparisons.

Next, we examined whether the cleavage products produced by UNG-1 are needed to induce Pvl, i.e., whether AP sites are transformed into 3′-blocked SSB via the AP lyase activity of NTH-1. The percent of *exo-3* mutants with Pvl was comparable to that of *nth-1;exo-3* mutants (Fig. 7b), suggesting that NTH-1 is not necessary for *dut-1 (RNAi)*-induced Pvl.

### The increase in *dut-1 (RNAi)*-induced Pvl in the *exo-3* mutants is driven by CHK-2

We hypothesized that the UNG-1-dependent increase in *dut-1 (RNAi)*-induced Pvl in the *exo-3* mutants can occur via DNA checkpoint activation driven by cleavage products produced by UNG-1 in addition to developmental delay. It has also been reported that *dut-1 (RNAi)*-induced Pvl is caused by the checkpoint kinase CLK-2^27^. Therefore, we examined whether the increase in *dut-1 (RNAi)*-induced Pvl in the *exo-3* mutants was dependent on CHK-2 and CLK-2. Although 49% of *exo-3;control (RNAi)* worms had *dut-1 (RNAi)*-induced Pvl, only 10% of the *exo-3;chk-2 (RNAi)* worms had it (Fig. 6b). On the other hand, the proportion of *exo-3;clk-2 (RNAi)* worms with Pvl was almost the same (44%) as that of the *exo-3;control (RNAi)* worms. Therefore, the knockdown of *chk-2* rescued the increase in *dut-1 (RNAi)*-induced Pvl in the *exo-3* mutants, as observed for developmental delay. These results suggest that the increase in *dut-1 (RNAi)*-induced Pvl in the *exo-3* mutants occurs via CHK-2 expression.

### Oxidative DNA damaging agents enhanced the proportion of Pvl in the *exo-3* mutants only when combined with *dut-1 (RNAi)*

To investigate other DNA damaging agents that cause an increase in Pvl in the *exo-3* mutants, we evaluated whether Pvl formation is enhanced by oxidative DNA damaging agents such as *ndx-1 (RNAi)*, *ndx-2 (RNAi)* and methyl viologen (MV). NDX-1 and NDX-2 hydrolyze 8-oxo-dGDP into 8-oxo-dGMP^24,28^. Accordingly, *ndx-1 (RNAi)* and *ndx-2 (RNAi)* lead to an increase in 8-oxo-dGDP in the nucleotide pool. The presence of 8-oxo-dGDP reduces the 8-oxo-dGTPase activity of NDX-4, causing 8-oxo-dGTP to accumulate in the pool^24^. 8-oxo-dGTP is incorporated to DNA during DNA replication, resulting in the accumulation of 8-oxoG in DNA^24,28^. MV generates superoxide radicals, which subsequently cause oxidative lesions in DNA^29^. However, single treatment with *ndx-1 (RNAi)*, *ndx-2 (RNAi)* or MV had no effect on the incidence of Pvl in the *exo-3* mutants. (data not shown). Next, we examined whether, when combined with the *dut-1 (RNAi)* treatment, each oxidative DNA damaging agent further enhanced the increase in Pvl in the *exo-3* mutants (Fig. 7a). Each treatment tested enhanced the increase in *dut-1 (RNAi)*-induced Pvl in the *exo-3* mutants by approximately 10% (Fig. 7b), suggesting that oxidative lesions in DNA can also cause an increase in Pvl.

In *in vitro* experiments, NTH-1 was found to possess weak DNA glycosylase activity toward 8-oxoG in DNA, in addition to its much higher activity toward oxidative pyrimidine lesions^30^. Thus, we suspect that the higher increase in *dut-1 (RNAi)*-induced Pvl by the additional oxidative agents depends on the activity of NTH-1. Although we confirmed the dependency of the phenotype on NTH-1, NTH-1 deficiency did not alter the proportion of worms exhibiting Pvl (Fig. 7b).

## Discussion

**Figure 8:**
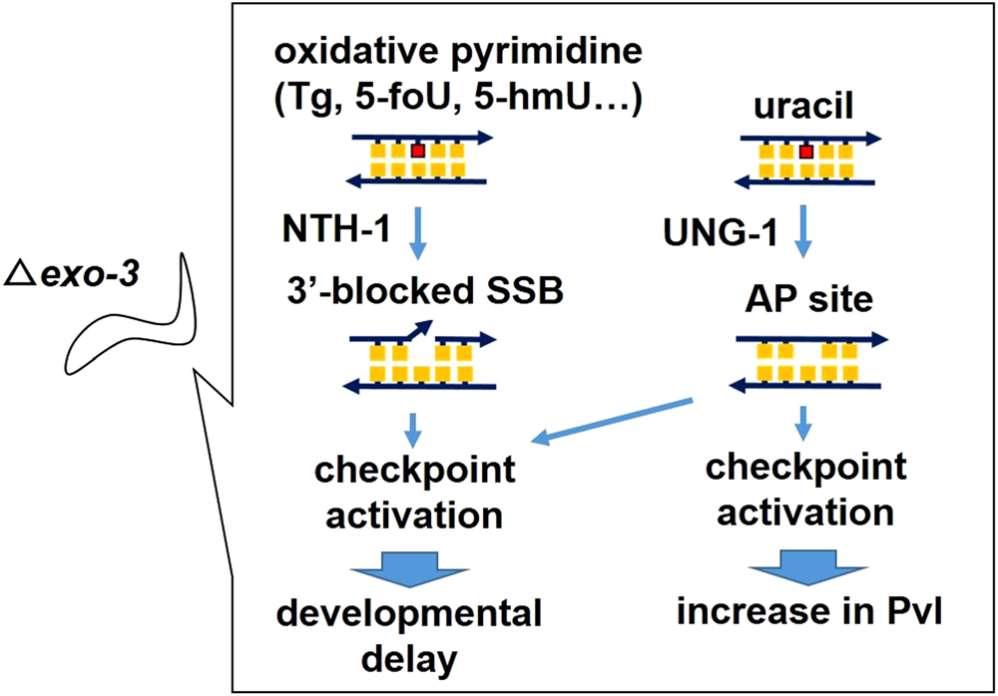
A model for the mechanisms of developmental delay and the increase in Pvl in the *exo-3* mutant worms. Cleavage products produced by either NTH-1 or UNG-1 can cause checkpoint activation, leading to developmental delay when worms lack EXO-3, but not when worms lack APN-1. Moreover, UNG-1-inducing checkpoint activation results in an increase in *dut-1 (RNAi)*-induced Pvl when worms lack EXO-3, but not when they lack APN-1.

In this study, we investigated the *in vivo* contribution of AP endonucleases from the larval to adult stages in *C*. *elegans* by evaluating development and vulval organogenesis in AP endonuclease gene mutants, and clarified that AP endonuclease EXO-3 deficiency causes developmental delay and an increased incidence of *dut-1 (RNAi)*-induced Pvl via DNA glycosylase-initiated checkpoint activation (Fig. 8).

The *exo-3* mutants demonstrated developmental delay, whereas the *apn-1* mutants did not (Fig. 1e), suggesting that EXO-3 has a more important role than APN-1 during development from the larval to adult stages.

The delay in the *exo-3* mutants was completely dependent on NTH-1 (Fig. 2), suggesting that it is caused by 3′-blocked SSB generated by NTH-1. We therefore hypothesized that AP sites cannot cause developmental delay in the *exo-3* mutants. However, MMS and NaHSO_3_ induced further developmental delay in the *exo-3* mutants (Fig. 3b,c), and we found that the delay by NaHSO_3_ was dependent on UNG-1 (Fig. 3c), suggesting that AP sites also caused developmental delay in the *exo-3* mutants, although the further delay may be caused by a mixture of both AP sites and cleaved AP sites with 3′-blocked SSB by NTH-1.

The developmental delay in the *exo-3* mutants was also due to CHK-2 (Fig. 4b). CHK-2 is an ortholog of mammalian Chk2, which is activated in response to several DNA damaging agents that cause DSB^31^, but there is no direct evidence that *C*. *elegans* CHK-2 is involved in a checkpoint mechanism driven by SSB. As 3′-blocked SSB can generate DSB during DNA replication^6^, as can AP sites^32^, it is possible that the resulting DSB activated the CHK-2 response, thereby leading to developmental delay. It was recently reported that ATM, which positively regulates Chk2 activity in response to DSB-generating agents^33,34^, is also activated by SSBs in human cells^35^. Thus, it is possible that 3′-blocked SSB directly activate CHK-2 via an ATM-1/CHK-2 pathway.

The *exo-3* mutants exhibited an increased incidence of *dut-1 (RNAi)*-induced Pvl (Fig. 5d), but the *apn-1* mutants did not, suggesting that EXO-3 has a more important role than APN-1 in vulval organogenesis and development. The increase in Pvl in the *exo-3* mutants was completely dependent on UNG-1 (Fig. 6a). This suggests that Pvl is caused by UNG-1-generating AP sites. The increase in Pvl occurred irrespective of NTH-1 (Fig. 7b), suggesting that the transformation of AP sites into 3′-blocked SSB by NTH-1 is not needed to induce the increase in Pvl.

Dengg *et al*. previously reported that *dut-1 (RNAi)*-induced Pvl occurs via the checkpoint kinase CLK-2^27^. CLK-2 may be activated by replication fork collapse-mediated DSB because they found that *dut-1(RNAi)* enhances the accumulation of RPA-1, ATL-1 (ATR ortholog in *C*. *elegans*) and RAD-51 in mitotic germ cells based on UNG-1 activity^27^. In this study, we tried to clarify whether the increase in *dut-1 (RNAi)*-induced Pvl in the *exo-3* mutants was dependent on CLK-2, but knockdown of CLK-2 had no effect on the increased incidence of Pvl (Fig. 6b). This discrepancy may result from the methods used to compromise CLK-2 function. Dengg *et al*. used *clk-2* mutant worms, whereas we used *clk-2 (RNAi)* worms, as reported previously^24^. Instead of CLK-2, another checkpoint kinase, CHK-2, was found to be a causal factor of the increase in Pvl (Fig. 6b). It is possible that a mechanism similar to CLK-2 activation causes Pvl formation through CHK-2 in the *exo-3* mutants.

The increase in *dut-1 (RNAi)*-induced Pvl in the *exo-3* mutants was further enhanced by oxidative DNA damaging agents such as *ndx-1 (RNAi)*, *ndx-2 (RNAi)* and MV (Fig. 7b). The damaging agents may have only caused Pvl in the *exo-3* mutants when combined with *dut-1 (RNAi)* because of a threshold of DNA lesions needed for Pvl. Combining each oxidative damaging agent with *dut-1 (RNAi)* results in more DNA lesions containing 8-oxoG than single *dut-1 (RNAi)* treatment. However, a direct homolog of MutM or OGG1 cannot be detected in *C*. *elegans*. As a candidate protein to incise 8-oxoG in DNA *in vivo* in *C*. *elegans*, we examined NTH-1. However, the effects of *ndx-1* and *ndx-2* knockdown on Pvl were independent of NTH-1 (Fig. 7b). A novel DNA glycosylase that can incise 8-oxoG may be responsible for this phenotype, but further studies are needed.

Pvl is formed by prevention of ras/notch/wnt signaling pathway^36–38^. We demonstrated that the checkpoint activation caused by UNG-1 results in the induction of Pvl, while the downstream pathway to induce Pvl still remains unclear. Thus, it is possible that checkpoint activation affects Pvl induction through modulation of other pathways such as ras/notch/wnt signaling pathways. However, there is no evidence of the link between checkpoint activation and such signaling pathways.

It is reasonable that checkpoint activation causes developmental delay, while it seems paradoxical that the activation also causes Pvl induction. The reason for the point is that developmental delay induced by checkpoint activation is predicted to be a mechanism for preventing mutagenesis, which provides worms beneficial effects. In contrast, Pvl caused by the activation seems to have no valuable effects. However, it is probable that the induction of Pvl, resulting in egg-laying-defective (Egl) worms^27^, is a mechanism for preventing the accumulation of mutations in the next generation.

This study demonstrated that EXO-3 prevents DNA glycosylase-initiated checkpoint activation in order for worms to grow at a normal speed and with normal vulva. Although there have been many studies reported a correlation between BER and biological phenomena, such as carcinogenesis and aging, few studies demonstrating causation have been conducted^39,40^. Due to the availability of *C*. *elegans* mutants and the characteristics of *C*. *elegans* mutants lacking AP endonucleases enabling them to mature past embryonic stages and produce the next generation, we were able to examine causal relationships between BER and many biological phenomena throughout life. Further studies on AP endonucleases using *C*. *elegans* will provide more detailed information about the *in vivo* roles of AP endonucleases in multicellular organisms.

## Methods

### *C*. *elegans* strains and culture conditions

The wild-type strain (Bristol N2) and RB877 [*nth-1(ok724)* III]^30^ were obtained from the Caenorhabditis Genetics Center, University of Minnesota. TM4374 [*exo-3(tm4374)* I]^41^, TM6691 [*apn-1(tm6691)* II]^14^ and TM2862 [*ung-1(tm2862)* III]^42^ were supplied by the National Bioresource Project for the Nematode (Tokyo Women’s Medical College). All mutants were backcrossed with N2 worms at least 3 times to remove background mutations, and the homozygous mutant progeny were used in the following experiments. The *apn-1;exo-3*, *nth-1;exo-3*, *ung-1;exo-3* and *nth-1;ung-1;exo-3* mutants were generated by crossing each strain. Deletion in the *exo-3*, *apn-1*, *nth-1* and *ung-1* genes was confirmed by PCR utilizing the same 2 primer pairs from a previous study^14^. In general, worms were cultured at 20 °C on NGM agar plates containing 0.3% (w/v) NaCl, 0.25% (w/v) polypeptone, 0.005% (w/v) cholesterol, 1 mM MgSO_4_, 1 mM CaCl_2_ and 25 mM potassium phosphate, at pH 6.0 with 0.17% (w/v) agar and a lawn of *Escherichia coli* (*E*. *coli*) OP50.

### Synchronization of worms

To obtain synchronized eggs, we made gravid adult worms lay eggs for approximately 2 hours on the same plates used for subsequent assays. Synchronized eggs were transferred to new plates for each assay.

### Bacteria-mediated RNA interference

For knockdown experiments, we used a well-established RNA interference (RNAi) method^43,44^. *C*. *elegans dut-1*, *ndx-1*, *ndx-2*, *chk-2 (Y60A3A*.*12)* and *clk-2 (C07H6*.*6)* complementary DNA (cDNA) clones were amplified by PCR from a cDNA library using the same 2 primer pairs from a previous study^24,28^. The amplified PCR products were subcloned into the plasmid L4440 for bacteria-mediated feeding RNAi (RNAi plasmid). Double RNAi experiments were performed by mixing an equal amount of overnight cultures of *E*. *coli* HT115 (DE3) that had been transformed with the respective RNAi plasmids, and then plating the mixture on NGM plates containing 0.1 mM IPTG and 100 μg/ml ampicillin (RNAi plates)^24^. As a negative control for RNAi, the transformant harboring L4440 was used.

### Measurement of developmental speed

To assay the effects of AP endonuclease deficiency on larval development, synchronized eggs were placed on normal NGM plates, NGM plates containing 0.94 mM MMS or 10 mM NaHSO_3_, or RNAi plates. After incubation at 20 °C for 3, 3.5 (MMS) or 4 (NaHSO_3_) days, developmental stages of surviving worms were assessed based on vulval morphology and brooding of eggs to distinguish young adult worms from gravid adult worms. The proportion of worms at each developmental stage among the total number of surviving worms was calculated.

### Measurement of the proportion of Pvl worms

To assay the effects of AP endonuclease deficiency on organogenesis, synchronized eggs were placed on *dut-1 (RNAi)* plates (plus additional RNAi and 0.1 mM methyl viologen (MV), if necessary). After incubation at 20 °C for 4 days, the numbers of total adult worms and Pvl worms were counted, and the proportion of Pvl worms among adult worms was calculated. Significance was determined using one-way ANOVA with Tukey’s test for multiple comparisons.

### Microscopy

Observation and imaging of *C*. *elegans* were performed using an OLYMPUS SZX16 microscope (OLYMPUS, Japan).

### Real-time PCR analysis

Worms (N2 at 0, 24, 48, 60 or 72 hr) were collected and lysed using TriPure isolation reagent (Roche, Basel, Switzerland). Then, total RNA was extracted from the above supernatant with NucleoSpin RNA (Takara Bio, Shiga, Japan), as recommended by the manufacturer. First-strand cDNA was synthesized from total RNA using oligo-dT primer and ReverTra Ace (Toyobo, Osaka, Japan). Real-Time PCR was performed using Light-Cycler 96 (Roche) with THUNDERBIRD SYBER qPCR Mix (Toyobo, Osaka, Japan). The thermal cycler conditions were as follows: an initial denaturation step at 95 °C for 60 sec, followed by 45 three-step PCR cycles of 95 °C for 15 sec, 60 °C for 30 sec and 72 °C for 45 sec. Gene amplification specificity was confirmed by melting curve analysis. The following are the primer sequences: APN1, 5′-GCTATCAGGAAATTGAAGCA-3′ and 5′-TCCAGTTTAGAGGTTTCTTC-3; EXO3, 5′-CGGAGATGGAGGAGACGTTTA-3′ and 5′-TCTGGGTCACCGATTCCTTTG-3′; Y45F10D.4, 5′-CGAGAACCCGCGAAATGTCGGA-3′ 5′-GCCTCATCTTCCCTGGCAACCG-3′. The two-tailed Student’s t-test was used for statistical analysis.

## Data Availability

The datasets generated during and/or analyzed during the current study are available from the corresponding author on reasonable request.
